# Explanatory AI Predicts the Diet Adopted Based on Nutritional and Lifestyle Habits in the Spanish Population

**DOI:** 10.3390/ejihpe15020011

**Published:** 2025-01-24

**Authors:** Elena Sandri, Germán Cerdá Olmedo, Michela Piredda, Lisa Ursula Werner, Vincenzo Dentamaro

**Affiliations:** 1 Faculty of Medicine and Health Sciences, Catholic University of Valencia San Vicente Mártir, c/Quevedo, 2, 46001 Valencia, Spain; elena.sandri@ucv.es (E.S.); german.cerda@ucv.es (G.C.O.); 2 Doctoral School, Catholic University of Valencia San Vicente Mártir, c/Quevedo 2, 46001 Valencia, Spain; 3 Department of Medicine and Surgery, Research Unit Nursing Science, Università Campus Bio-Medico di Roma, Via Alvaro del Portillo, 21-00128 Rome, Italy; 4 Faculty of Teaching and Science of Education, Catholic University of Valencia San Vicente Mártir, c/Quevedo, 2, 46001 Valencia, Spain; lu.werner@ucv.es; 5 Department of Computer Science, University of Bari Aldo Moro, Via E. Orabona, 4, 70125 Bari, Italy; vincenzodentamaro@gmail.com

**Keywords:** machine learning, deep learning, diet, food, and nutrition, healthy lifestyle, Spain

## Abstract

This study used Explainable Artificial Intelligence (XAI) with SHapley Additive exPlanations (SHAP) to examine dietary and lifestyle habits in the Spanish population and identify key diet predictors. A cross-sectional design was used, employing the validated NutSo-HH scale to gather data on nutrition, lifestyle, and socio-demographic factors. The CatBoost method combined with SHAP was applied. The sample included 22,181 Spanish adults: 17,573 followed the Mediterranean diet, 1425 were vegetarians, 365 were vegans, and 1018 practiced intermittent fasting. Fish consumption was the strongest dietary indicator, with vegans abstaining and some vegetarians consuming it occasionally. Age influenced diet: younger individuals preferred vegan/vegetarian diets, while older adults adhered to the Mediterranean diet. Vegans and vegetarians consumed less junk food, and intermittent fasters were more physically active. The model effectively predicts the Mediterranean diet but struggles with others due to sample imbalance, highlighting the need for larger studies on plant-based and intermittent fasting diets.

## 1. Introduction

The type of diet people adopt and their lifestyle habits have a significant impact on health (Kandel, 2019; Cena & Calder, 2020). A balanced and healthy diet provides the essential nutrients the body needs to function optimally (Cena & Calder, 2020), and helps to maintain a healthy weight (Lim, 2018) and to prevent the onset of disease (Diab et al., 2023; Neuhouser, 2019; Gropper, 2023). Healthy sleep habits improve cognitive function (Khan & Al-Jahdali, 2023), strengthen physical and mental health (Tyagi et al., 2023), and regulate metabolism and weight (Cooper et al., 2018). In addition, adequate rest contributes to a longer life and reduces stress (Mazzotti et al., 2014). Regular physical activity strengthens muscles and bones (Hillsdon & Foster, 2018), improves cardiovascular health (Pinckard et al., 2019), helps maintain a healthy weight (Goldberg & King, 2007), promotes mental well-being, and improves mood (Mahindru et al., 2023), reducing the risk of chronic diseases (Usmani et al., 2023). Finally, exercise increases energy (Puetz, 2006), contributing to a better overall quality of life (Marquez et al., 2020). Therefore, the combination of a healthy diet and good lifestyle habits can have positive synergistic effects.

In Spain, the dietary pattern traditionally recognised as Mediterranean, characterised by high consumption of fruit and vegetables, use of olive oil as the main source of fat, reduced consumption of meat and dairy products, and moderate consumption of red wine, has stood out for its numerous health benefits (Guasch-Ferré & Willett, 2021; Shen et al., 2015; Dominguez et al., 2023). However, in recent years, its prevalence has declined while other dietary patterns, such as plant-based diets (PBDs), have gained ground in the population (Abellán Alemán et al., 2016; Herrera-Ramos et al., 2023).

PBDs are characterised by a reduced consumption of foods of animal origin (Acevedo Cantero et al., 2023; Euromonitor International, 2024). The most widely followed PBDs are the vegetarian diet (VD), which excludes some animal-based foods from the dietary plan, and the vegan diet (VG), which eliminates the consumption of all animal products. Another noteworthy dietetic behaviour is intermittent calorie restriction (ICR) or intermittent fasting (IF), which involves alternating periods of significant energy restriction with regular energy intake (Rynders et al., 2019).

Recent studies have investigated the health impact of these new dietary patterns (Yokoyama et al., 2014; Tantamango-Bartley et al., 2015; Ivanova et al., 2021; Welton et al., 2020; Yuan et al., 2022; Sandri et al., 2023a), and significant attention has also been devoted to understanding the motivations behind the adoption of vegetarian and vegan lifestyles (North et al., 2021; Miki et al., 2020), as well as intermittent fasting practices (Ganesan et al., 2018). However, studies quantifying the associations between a population’s dietary preferences and its social and lifestyle behaviours, as well as their connections with socio-demographic determinants are still lacking.

The field of health and social and lifestyle habits is broad and multifaceted, which often makes it difficult to understand in depth which variables most influence health, which factors determine one dietary choice over another, or which conditioning factors affect social and lifestyle habits and to what extent (Short & Mollborn, 2015). To address this complexity, a novel and increasingly popular solution is to use Artificial Intelligence (AI) (Bini, 2018).

Contemporary AI tools can achieve remarkable accuracy and help enormously in the context of medicine and healthcare in general, yet they can be complex to understand (Prokhorenkova et al., 2017; Dentamaro et al., 2024; Shen et al., 2017). This is particularly true for models known as “black-box”, whose internal workings are opaque or hidden even to experienced scientists. To address this, a new branch of Artificial Intelligence has been developed: Explainable Artificial Intelligence (XAI) (Gunning et al., 2019). XAI encompasses a set of tools and frameworks designed to help users comprehend and interpret the predictions made by machine learning (ML) models (Palar et al., 2023).

The central hypothesis underpinning this research is that there is a direct relationship between the dietary patterns adopted by individuals and their nutritional habits, social behaviours, and lifestyle choices. This relationship suggests that dietary practices do not exist in isolation but are deeply interconnected with broader social and cultural norms, as well as individual routines and health-related behaviours. Understanding these dynamics can provide valuable insights into how social and lifestyle factors influence dietary decisions, shaping public health outcomes and nutritional well-being.

This analysis aimed to apply XAI (specifically, an explainability method called SHAP (SHapley Additive exPlanations) to a dataset on eating and lifestyle habits of the Spanish population to identify the strongest predictors for certain diets.

## 2. Materials and Methods

### 2.1. Type of Study and Sampling

A cross-sectional study was conducted to describe the population residing in Spain aged 18 and older.

### 2.2. Inclusion and Exclusion Criteria

Inclusion criteria:
  - Individuals aged 18 years or older.
  - Spanish citizens.
  - Residents of Spain.
Exclusion criteria:
  - Individuals with chronic conditions could influence their dietary habits.
  - Participants experiencing temporary circumstances during the survey period that disrupted their usual diet, such as hospitalization, incarceration, or similar situations.

### 2.3. Ethical Approval

The research adhered to the ethical principles outlined in the Declaration of Helsinki (World Medical Association, 2013) and received approval from the Research Ethics Committee of the Catholic University of Valencia (approval code UCV/2019-2020/152, on 18 June 2020). Informed consent was obtained from all participants prior to their involvement in the study.

### 2.4. Instrument

The data collection instrument utilized in this study was the Nutritional and Social Healthy Habits Scale (NutSo-HH) (Sandri et al., 2024c). The instrument’s development and psychometric testing adhered to stringent methodological standards. An expert panel (N = 7), including a nutritionist, two family doctors, two psychologists, a social educator, and a communication expert, evaluated the content validity. Face validity was assessed through a pilot study involving 53 individuals with characteristics akin to the study population.

The scale comprises 23 items designed to evaluate various nutritional and lifestyle aspects, such as the frequency of consumption of specific foods and beverages, alcohol use, tobacco habits, and rest patterns, among other factors. Additionally, a section of the questionnaire was dedicated to investigating potential symptoms of eating disorders. The questionnaire also gathered information on self-perceived health status, diet-related illnesses, eating disorder symptoms, nutritional habits, food consumption frequency, sedentary behaviours, physical activity duration, and health-related social habits such as sleep patterns, smoking, and alcohol consumption. Moreover, the questionnaire explores socio-demographic variables including sex, age, municipality (the variable distinguishes the size of the municipality of residence in three categories: small (≤2000 inhabitants); medium (2000 < inhabitants ≤ 10,000); large (>10,000 inhabitants)), type of residence, home (variable discriminates whether a respondent lives in the family home or outside), alone (the variable discriminates whether a respondent lives alone or with other people), education level, and income. It also covers anthropometric variables such as weight and height, type of diet, and other relevant health variables to provide a comprehensive assessment of the participants’ nutritional and lifestyle habits. Most variables are qualitative, allowing multiple-choice and frequency range selections. Continuous quantitative variables include age, weight, height, and weekly minutes of sports. Discrete quantitative variables, such as self-reported health levels, follow a Likert scale format. Table A1 in Appendix A shows in detail the list of variables analysed and their typology.

### 2.5. Data Collection

The survey was hosted online and distributed via non-probabilistic snowball sampling, mainly through social media platforms like Instagram, LinkedIn, Twitter, WhatsApp, and Facebook. An Instagram account, @elretonutricional, was created to engage professionals and influencers.

To reach segments of the population less familiar with social networks, a physical dissemination of the questionnaire was also conducted. For this purpose, associations and establishments across Spain, selected for their diverse audiences, were contacted via email. After providing a detailed explanation of the project, those willing to collaborate in the dissemination were sent a poster featuring a QR code. They were asked to display the poster in their establishments or share it with their members. The poster presented the project in a concise and engaging manner, enabling participants to easily and quickly complete the online survey by scanning the QR code.

Data collection spanned from August 2020 to November 2021, ensuring a broad and diverse sample.

Before starting the data analysis with the machine learning methods, the obtained data were downloaded into a Microsoft Excel file in order to prepare it. Surveys that did not meet the inclusion criteria and those with incomplete or erroneous data (e.g., responses with a 14 kg/m^2^ < body mass index (BMI) < 40 kg/m^2^) were removed. Finally, the variables were categorised.

### 2.6. Grouping and Categorisation of Variables

**Figure 1:**
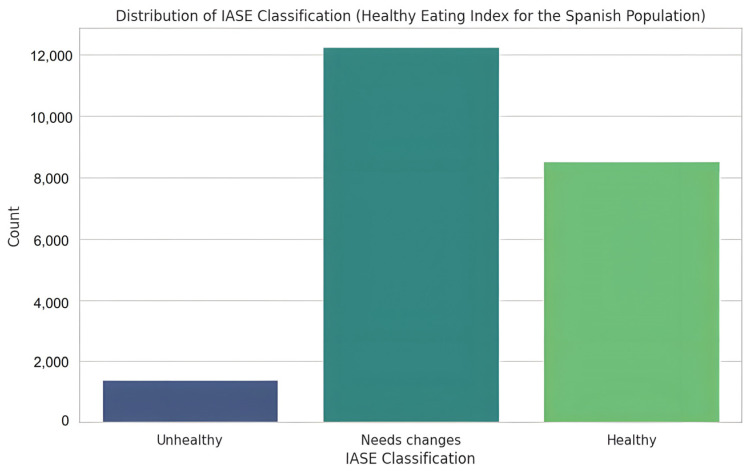
Classes distribution with respect to IASE classification.

Some of the food frequency variables (fruit, vegetables, meat, dairy, cereals, pulses and soft drinks) were utilized to calculate the Healthy Eating Index for the Spanish population (IASE) (Índice de Alimentación Saludable de la población Española), based on a validated condensed version by (Norte Navarro & Ortiz Moncada, 2011). Based on the IASE score, nutritional habits were classified into three categories: “Healthy” (58.4 < IASE ≤ 73), “Needs changes” (36.5 ≤ IASE ≤ 58.4), and “Unhealthy” (IASE < 36.5). A detailed description of how the scale has been adapted can be found in a previous article (Sandri et al., 2023b, 2024b). Figure 1 shows the distribution of the population with respect to the IASE score obtained.

Nutritional variables not addressed by the IASE and lifestyle variables were categorized on a Likert scale ranging from 1 to 4 points, except for signs of eating disorders, which used a Likert scale of 1 to 6 following the same criteria as in the previous article (Sandri et al., 2024a, 2024d). Finally, body mass index (BMI) and minutes of sports were used as a numerical variable.

The variable to be predicted was Special Diet, containing the following classes: Mediterranean diet, vegan, intermittent fasting, ovolactovegetarian, flexitarian diet, lactovegetarian, ovovegetarian. The last 4 categories, which are variants of the vegetarian category, were all grouped within this category.

### 2.7. Comparison and Selection of Machine Learning Algorithms

Machine learning algorithms are not universally applicable; multidimensional experimental studies involving multiple classification algorithms and model testing methods are necessary to select the most suitable approach. Initially four classification models were considered, specifically: Random Forest, an ensemble model that combines the output of multiple decision trees to reach a single result (Breiman, 2001). Histogram-based Gradient Boosting (Nhat-Duc & Van-Duc, 2023), an ensemble model based on the Gradient Boosting technique, which is a model that operates sequentially by combining the predictions of, typically, decision trees. XGBoost (Chen & Guestrin, 2016), a short for eXtreme Gradient Boosting, is one of the most used ensemble models due to its speed. CatBoost, short for Categorical Boosting (Dorogush et al., 2018; Hancock & Khoshgoftaar, 2020), is a gradient boosting implementation optimized to deal with categorical features. All these models were run and the results were compared to select the best one that finally was CatBoost.

Developed by Yandex (2017), CatBoost stands out in several key areas as delivers high-quality results without parameter tuning, supports categorical features, implements GPU acceleration or provides fast predictions. CatBoost addresses a common limitation of other decision tree-based methods, which typically require categorical string variables to be converted into numerical values through pre-processing. It can directly handle a mix of categorical and non-categorical explanatory variables without needing such pre-processing. CatBoost’s encoding method, called Ordered Encoding, uses target statistics from all preceding rows to determine a value for replacing the categorical feature (Hancock & Khoshgoftaar, 2020). This unique capability was the main reason for choosing this model over others.

### 2.8. SHapley Additive exPlanations (SHAP) Approach

For the interpretation of the results obtained with the CatBoost’s method, the SHAP approach was used. SHAP is based on the so-called SHAP values that are used to explain the output of a ML model (Lundberg & Lee, 2017). SHAP values are based on the Shapley values from game theory and assign an importance value to each feature in a model (Ui, 2000). Positive SHAP values positively impact the prediction, while negative ones have a negative impact (Rodríguez-Pérez & Bajorath, 2020).

While model-agnostic, SHAP is especially good for tree ensemble methods (Lundberg et al., 2020) (like CatBoost). The most important properties of SHAP Values are:

(1) Additivity SHAP values are additive, meaning that the contribution of each feature to the final prediction can be computed independently and then summed up.
(2) Local accuracy SHAP values sum up to the difference between the expected model output and the actual output for a given input.
(3) Missingness SHAP values are zero for features that are missing or irrelevant to a prediction.
(4) Consistency SHAP values only change when the model is modified if the contribution of a feature changes.

SHAP offers several explainers optimized for certain models and has a powerful API to create plots that can guide the analysis of the results (Palar et al., 2023).

The most used SHAP plots are:

(1) Dependency plot: A scatter plot that shows the effect a single feature has on the predictions made by the model.
(2) Force plot: Shows how features contribute to the model’s prediction for a specific observation.
(3) Waterfall plot: Used to gain insights into how features contribute to a single prediction. Starting from a base value, each row shows how the positive (red) or negative (blue) contribution of each feature moves the value from the expected model output (E[f(x)]) to the model output for that prediction (f(x)).
(4) Decision plot: Provides insights into how each feature contributes to the model’s prediction for a specific instance and how a model arrives at its predictions. It is similar to a force plot, but it is more useful when there are a large number of significant features involved due to the direction in which the plot goes.
(5) Summary plot: Provides insights into how each feature contributes to the output of the model from a global point of view. Its components are:
  - feature importance: represented on the Y-axis with the most important features at the top and the least important at the bottom;
  - SHAP values: represented on the X-axis, they can be positive (to the right) or negative (to the left);
  - feature value: represented by the colour scale that can be seen on the right of the plot.

Moreover, the densest portions are “taller” than the others.

The summary plot was used to obtain the results of this study as it provided the most useful information for our purposes.

### 2.9. Approach for Interpretable Machine Learning

The experiment was conducted using a systematic and rigorous approach, ensuring the validity and reliability of the results. The following steps were meticulously executed:

- Data Preparation:
  ○ Data Loading and Cleaning: The dataset was first read into the analysis environment. The target feature, which indicates the dietary pattern, and the sex variable were removed since the dataset only included female participants. This step was crucial to prevent any potential bias in the model that could arise from gender-specific dietary habits.
  ○ Unification of Classes: The various vegetarian dietary patterns (ovolactovegetarian, flexitarian, lactovegetarian, ovovegetarian) were unified into a single ’Vegetarian’ class. This decision was based on the rationale that these diets share significant similarities and consolidating them would enhance the model’s ability to generalize across different vegetarian diets.
  ○ Handling Missing Data: Any rows with missing values (NaNs) were removed to ensure that the dataset was complete. This step was critical to avoid introducing biases or inaccuracies in the model due to incomplete data.

In order to mitigate the inherent class imbalance observed within the dataset, a thorough investigation into various resampling methodologies was undertaken. The initial class distribution of the IASE Classification, as depicted in Figure 1, reveals a notable disparity; specifically, 1407 samples (constituting 6.3%) were categorized as “Unhealthy”, 12,252 samples (representing 55.2%) were classified as “Needs changes”, and 8522 samples (accounting for 38.4%) were designated as “Healthy”. Three distinct strategies were evaluated to address this imbalance: the application of weighted classes, the implementation of targeted sampling, and the utilization of SMOTENC (Synthetic Minority Over-sampling Technique for Nominal and Continuous features). The weighted classes approach incorporated empirically derived weights of 5.255, 0.603, and 0.868 for the unhealthy, needs changes, and healthy classes, respectively; these weights were calculated based on the inverse class frequencies. While both the weighted classes and targeted sampling techniques demonstrated improvements in the prediction of the minority class, SMOTENC exhibited superior performance, particularly in the preservation of the complex relationships between nominal and continuous features present within the dietary data. This advantage was particularly evident in maintaining the intricate correlations between nutritional components while generating synthetic samples for the underrepresented unhealthy class. The selection of SMOTENC was further substantiated by its capacity to maintain enhanced F1-scores across all classes when compared to alternative methodologies. The employment of SMOTENC, therefore, was deemed the most appropriate course of action given its demonstrated efficacy in addressing the class imbalance while preserving the integrity of the dataset’s inherent structure and relationships. This approach ensures a more robust and reliable analysis of the dietary data, mitigating the potential biases introduced by the initial class imbalance. The careful consideration of these factors led to the selection of SMOTENC as the preferred method for data preprocessing.

### 2.10. Model Selection and Optimization

For the task concerning dietary pattern classification, the fitting machine learning model’s selection necessitated a judicious assessment of the attributes inherent within our dataset, encompassing a mixture of categorical and numerical features, alongside the presence of class imbalance. A thorough assessment of differing methodologies was undertaken, with the eventual selection of CatBoost as our principal model. CatBoost, owing to its implementation of ordered boosting and the efficient processing it affords for categorical features, rendered it particularly apt for our nutritional data analysis endeavours.

Whilst CatBoost is well-regarded for the inherent robustness of its default parameter configurations, we did perform a grid search optimization, with focus placed upon pivotal hyperparameters that bear upon model complexity and the capacity for generalization. The ranges subjected to testing encompassed learning_rate ∈ {0.01, 0.05, 0.1, 0.3}, depth ∈ {4, 6, 8, 10}, and l2_leaf_reg ∈ {1, 3, 5, 7}. It is noteworthy that the default configuration (learning_rate = 0.1, depth = 6, l2_leaf_reg = 3) attained peak performance, manifesting a macro F1-score of 0.82, a balanced accuracy of 0.79, and a ROC-AUC of 0.85 when evaluated across all classes.

For the purpose of corroborating our selection of a model, the implementation of Google TabNet, (Arik & Pfister, 2021), a deep neural network architecture purpose-built for tabular data through the utilization of attention mechanisms, was undertaken. Notwithstanding its intricate design, TabNet achieved suboptimal outcomes (macro F1-score: 0.76, balanced accuracy: 0.73, ROC-AUC: 0.81), thereby reinforcing the recognized pre-eminence of tree-based ensembles for the classification of structured tabular data. This discrepancy in performance was especially conspicuous in the management of the complex interactions existing between dietary components and categorical variables representative of eating behaviours.

The ultimate CatBoost model demonstrated commendable performance when evaluated against all metrics, exhibiting class-specific F1-scores of 0.79 (unhealthy), 0.83 (needs changes), and 0.84 (healthy), adeptly navigating the intrinsic imbalance in class representation whilst upholding high predictive accuracy.

- Dataset Splitting:
  ○ The dataset was split into training and test sets using an 80-20 split ratio. This stratified split ensured that the test set was representative of the overall dataset, allowing for robust evaluation of the model’s performance. The rationale behind this split is to maintain enough data for training while preserving a portion for unbiased testing.
- Feature Standardization:
  ○ Z-score normalization (Z-points scaler) was applied to standardize the features.

This technique, which involves scaling the data to have a mean of zero and a standard deviation of one, was chosen to ensure that all features contributed equally to the model training, thereby preventing features with larger scales from dominating the learning process.

- Model Training:
  ○ Several machine learning models have been tested including Random Forest, Decision Trees, Feed Forward Neural Networks, and CatBoost. The best performing model was the CatBoost classifier due to its superior handling of categorical features and its ability to provide high-quality predictions without extensive parameter tuning. Additionally, its implementation of Ordered Encoding for categorical features significantly reduced the risk of overfitting.
- Model Testing:
  ○ The trained CatBoost model was then tested on the test set. Performance metrics, including precision, recall, and F1-score, were calculated and the confusion matrix was generated. These metrics provided a comprehensive evaluation of the model’s classification capabilities across different dietary classes.
- Interpretability with SHAP:
  ○ To interpret the model’s predictions, the TreeExplainer model from the SHAP package was utilized. SHAP values, which are grounded in game theory, were calculated to assign an importance value to each feature. This approach ensures transparency in the model’s decision-making process, allowing for a detailed understanding of how each feature influences the predictions.
  ○ SHAP Plots Generation:
    Several SHAP plots were generated to visualize the feature contributions:
    Summary plot: This plot provided a global view of feature importance, highlighting which features had the most significant impact on the model’s predictions.
    Dependency plot: This plot illustrated the relationship between individual features and the predicted output.
    Force plot and waterfall plot: These plots provided insights into the contributions of each feature to individual predictions, demonstrating how the model arrived at specific decisions.
- Oversampling with SMOTENC:
  ○ The Synthetic Minority Over-sampling Technique for Nominal and Continuous (SMOTENC) was applied to address class imbalance in the training set. SMOTENC generates synthetic samples for minority classes, balancing the dataset. This technique was particularly effective in improving the classification performance for underrepresented dietary patterns. Importantly, oversampling was only applied to the training set to ensure that the test set remained a valid benchmark for evaluating model performance.

**Figure 2:**
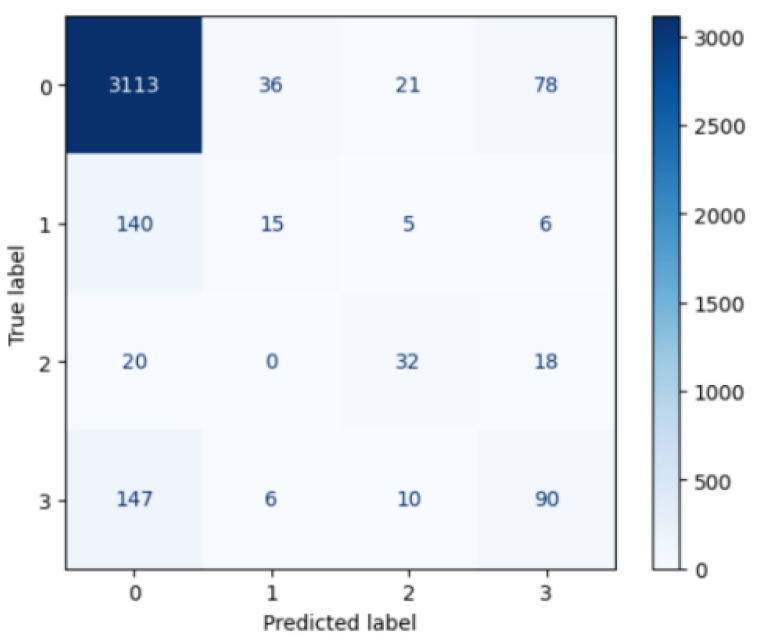
SMOTENC confusion matrix.

The application of these detailed and methodologically sound steps ensured the robustness and interpretability of the machine learning model, providing reliable insights into the dietary patterns of the study population (Table 1 and Figure 2).

Table 1 and Figure 2 reveal the performance of the CatBoost model in predicting different dietary patterns. For the Mediterranean diet, the model shows high precision (0.91), recall (0.96), and F1-score (0.93), indicating that it effectively identifies individuals following this diet with minimal false positives and negatives. This high performance is supported by a large sample size (3248), which ensures robust training data.

In contrast, the model performs poorly for intermittent fasting, with low precision (0.26), recall (0.09), and F1-score (0.13), suggesting that it struggles to accurately predict this diet, likely due to a small sample size (166) and resulting in high false positive and negative rates despite the use of SMOTENC. The performance for the vegan diet is moderate, with precision (0.47), recall (0.46), and F1-score (0.46), reflecting the high nonlinearity of the dataset in correctly identifying vegans due to a very small sample size (70). Similarly, for the vegetarian diet, the model shows moderate performance with precision (0.47), recall (0.36), and F1-score (0.40), indicating difficulties in prediction accuracy due to a limited sample size (253).

Overall, the model achieves an accuracy of 87%, which is acceptable and reflects the strong representation of the Mediterranean diet class. The macro-average precision (0.53), recall (0.47), and F1-score (0.48) indicate a moderate overall performance across all classes. These results, although not optimal, provide valuable insights and highlight the potential need for additional or more nuanced features to better capture the non-linearities and nuances necessary for accurately discerning specific dietary patterns, thereby enhancing our understanding and prediction of dietary habits.

## 3. Results

### 3.1. Socio-Demographic Characteristics of the Sample

Most respondents are women (80.8%), on average 34.9 years old (SD 11.7; range 18–89), with higher education (68.3%), and living in a city (79.3%). The income level is quite homogeneous, with a similar percentage of people with low income (43.8%) compared to people with medium–high income (47.7%). More details of the socio-demographic characteristics of the sample are provided in Table 2.

### 3.2. Summary Plots

**Figure 3:**
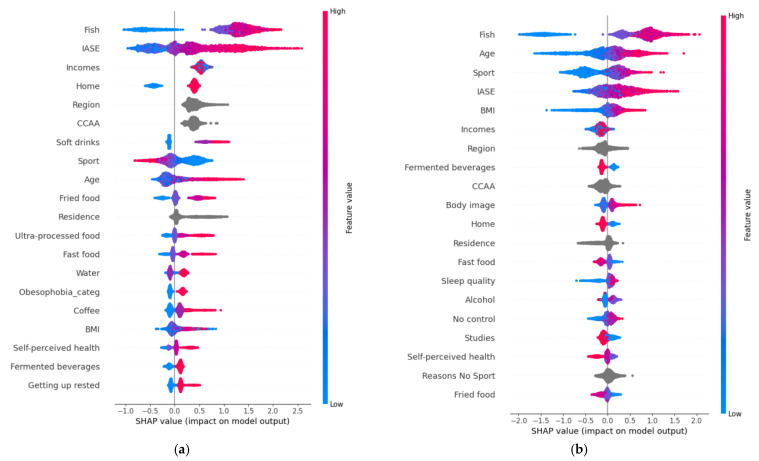

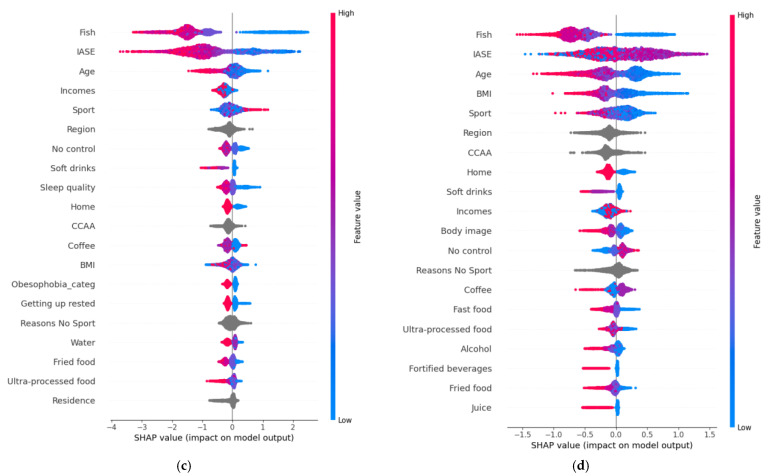
(**a**) Summary plot for Mediterranean diet class. (**b**) Summary plot for intermittent fasting diet class. (**c**) Summary plot for vegan diet class. (**d**) Summary plot for vegetarian diet class.

Figure 3a–d show the summary plot for the different diet categories. From the first summary plot (Figure 3a), key features influencing the Mediterranean diet include:

- Fish: A strong divide exists between non-consumers (negative influence) and consumers (positive influence).
- IASE: Higher values generally correlate with a greater likelihood of following the diet.
- Income: Though influential, values cluster between 0.2 and 0.5.
- Sport: Those practicing more sport tend to not follow this diet.
- Age: Older individuals are more likely to follow the Mediterranean diet.
- Junk Food: Non-consumers of junk food tend not to follow this diet.

Analysis of dependence plots reveals:

- Age: Confirms the tendency for older individuals to follow the diet, with a specific age where adherence increases. Younger people consume less fish.
- Body Image: Better self-image correlates with more controlled eating habits, though with low impact.
- BMI: A low BMI predicts adherence, especially in physically active individuals. Neutral BMI has minimal influence, but higher BMI positively impacts predictions.
- Sport: Less sport correlates with higher adherence.
- Eating Disorders: Anorexia nervosa positively impacts predictions, while binge eating has the opposite effect.
- Illness: Illnesses like diabetes and fructose intolerance increase adherence.
- CCAA (Autonomous Regions, Comunidades Autónomas): Catalans tend not to follow this diet.

Figure 3b provides the summary plot for the intermittent fasting diet category.

**Figure 4:**
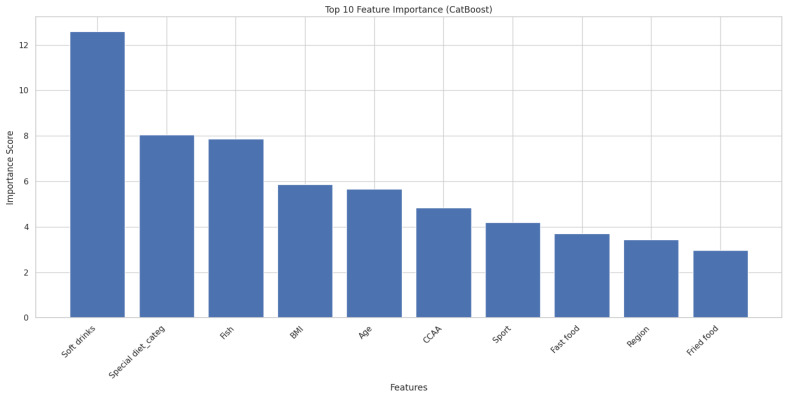
Top 10 feature importance for classifying IASE score.

Figure 4 show the top 10 feature importance for classifying IASE score.

From the summary plot (Figure 3b), the most influential features for the intermittent fasting diet include:

- Fish: Non-consumers tend not to follow this diet.
- Age: Younger people are less likely to follow the diet, while older individuals are more likely to adhere.
- Sport: Those who practice sport are more likely to follow this diet, though the group is small.
- BMI: Lower BMI negatively impacts predictions, while higher BMI positively influences adherence.
- Income: Similar influence as in the Mediterranean diet category.
- Home: Unclear impact, similar to the Mediterranean diet.
- Junk Food: Consumers are less likely to follow this diet, though the overall impact is minimal.
- Sleep Quality: Lower sleep quality correlates with a reduced likelihood of adherence.

Dependence plot analysis provides additional insights:

- Body Image: Minimal impact overall, but extreme values show a strong positive (high body image) or negative (low body image) influence.
- BMI: Neutral BMI has little effect. Two trends emerge: lower BMI decreases adherence probability, while higher BMI increases it.
- Sport: In general, less sport correlates with lower adherence, but exceptions exist.
- Fried Food: Lower fried food consumption predicts adherence. Among younger individuals, fried food strongly reduces adherence, but this effect diminishes with age.
- Sleep: Longer sleeping hours slightly decrease adherence, but better sleep quality significantly increases it.
- CCAA: Galicians are less likely to follow this diet.

These findings underscore lifestyle and demographic factors influencing adherence to the intermittent fasting diet.

From the summary plot (Figure 3c), the most influential features for following a vegan diet are:

- Fish: People who do not eat fish are more likely to follow a vegan diet.
- Sleep Quality: Poor sleep quality is associated with higher adherence.
- Age: Younger individuals are more likely to be vegan, while older people are less likely.
- No Control: Those with less control over their eating habits tend to follow the vegan diet.
- Sport: Non-athletic individuals are less likely to follow this diet.
- BMI: Higher BMI correlates with lower adherence.
- Sleeping Hours: People who sleep less are more likely to follow the diet.
- Ultra-Processed Food: Higher consumption of ultra-processed food decreases the likelihood of adherence.
- Obesophobia: Fear of obesity is a factor; those unconcerned about obesity tend not to follow the diet.

The dependence plots add the following insights:

- Age: Strong confirmation that younger individuals follow the vegan diet.
- BMI: As BMI increases, adherence decreases.
- Fried Food: Avoidance of fried food is linked to veganism; interestingly, some sport participants eat fried food and are vegan.
- Ultra-Processed Food: Minimal consumption strongly predicts adherence, while high consumption has a stronger negative impact.
- Sleeping Patterns: Less sleep correlates with adherence, and worse sleep quality strengthens this connection.
- Eating Disorders: Most vegans do not report eating disorders.
- Illness: Lactose intolerance significantly reduces the likelihood of following a vegan diet.
- CCAA: Catalonia and Galicia show the highest concentrations of vegans.

These findings highlight demographic, lifestyle, and health factors influencing vegan diet adherence.

From the summary plot (Figure 3d), the key factors influencing adherence to a vegetarian diet are:

- Fish: People who do not consume fish are more likely to follow a vegetarian diet.
- Age: Younger individuals are more likely to follow the diet, while older individuals are less likely.
- BMI: Higher BMI correlates with lower adherence, whereas those with a low BMI are more likely to follow the diet.
- Sport: Those who engage in sports are less likely to follow the diet.
- Sleeping Hours: Individuals who sleep less are more likely to follow the diet.
- Junk Food: Higher consumption of junk food is associated with lower adherence.
- Body Image: People with a lower body image and poorer self-perceived health tend to follow the diet.

From the dependence plots, additional insights include:

- Age: Younger people consistently show higher adherence.
- Body Image: Those with very negative self-perception are more likely to follow the diet, while those with a highly positive image are less likely.
- BMI: As BMI increases, adherence decreases.
- Junk Food: Individuals consuming more junk food tend not to follow the diet.
- Fish: Avoidance of fish strongly predicts vegetarianism.
- Eating Disorders: Most vegetarians do not report any diagnosed eating disorders.
- CCAA: Similar to veganism, Galicia and Catalonia have the highest concentrations of vegetarians.

These findings underscore the influence of dietary preferences, lifestyle choices, and self-perception on vegetarian diet adherence.

**Figure 5:**
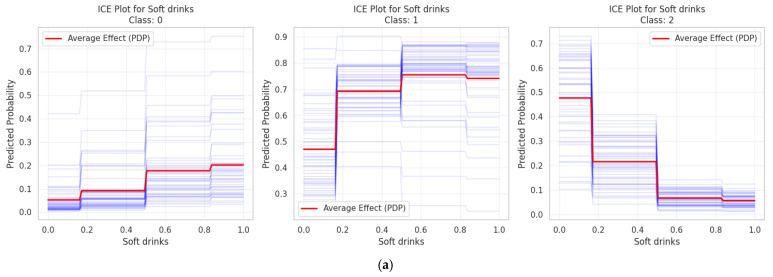

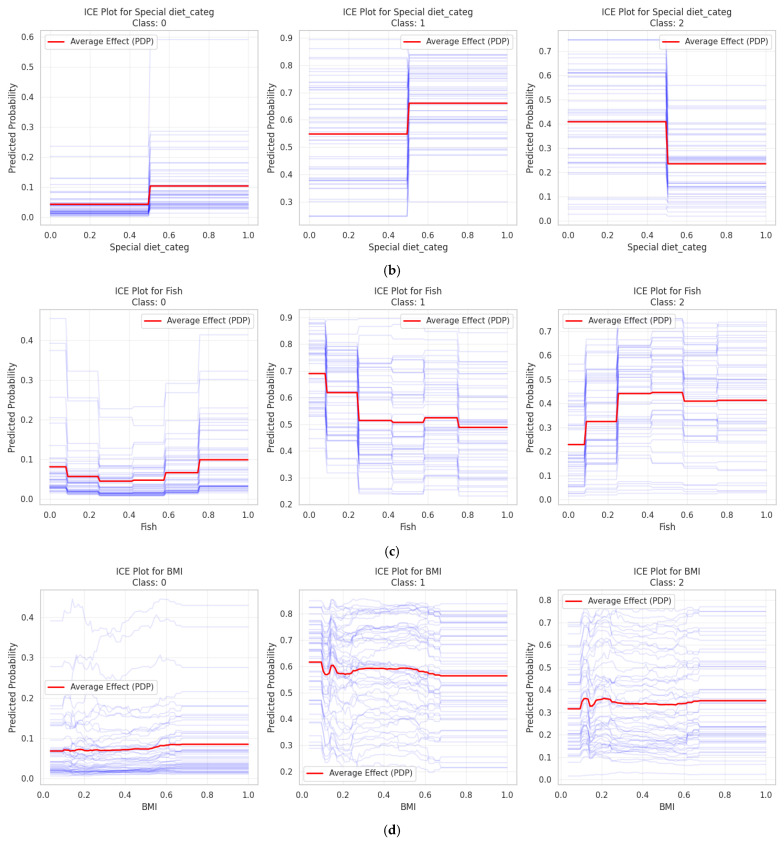

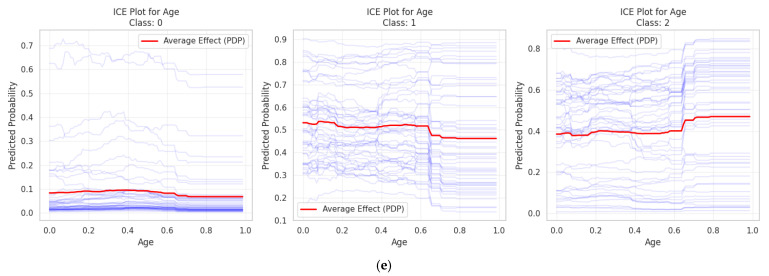
(**a**) ICE plot for soft drinks. (**b**) ICE plot for special diet. (**c**) ICE plot for fish. (**d**) ICE plot for BMI. (**e**) ICE plot for age.

Figure 5a–e show the ICE plots for different variables (Figure 5a: Soft Drinks; Figure 5b: Special Diet; Figure 5c: Fish; Figure 5d: BMI; Figure 5e: Age).

### 3.3. Analysis of the Decision Plots

#### 3.3.1. Discovering How the Model Decides the Class

To understand how the model predicts diet classes, the decision plot is analysed. Given the multi-class classification problem, SHAP’s multioutput_decision_plot is utilized to evaluate individual predictions.

**Figure 6:**
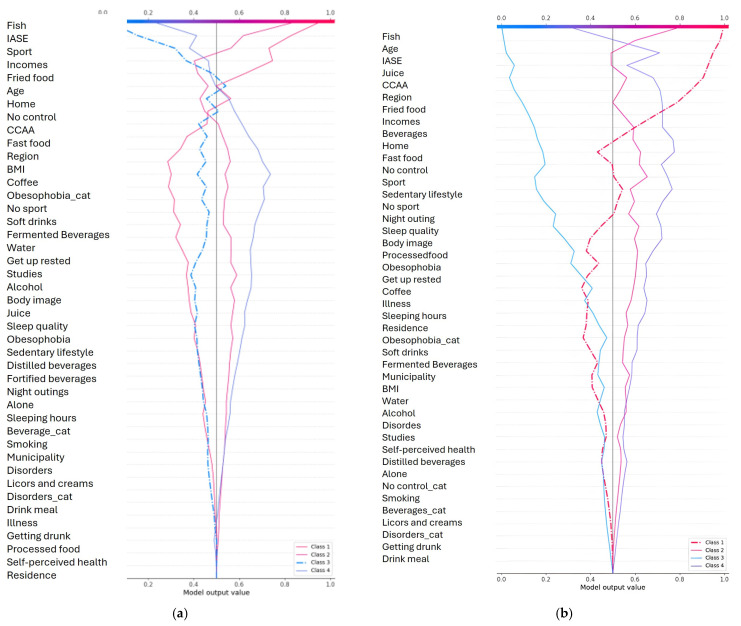

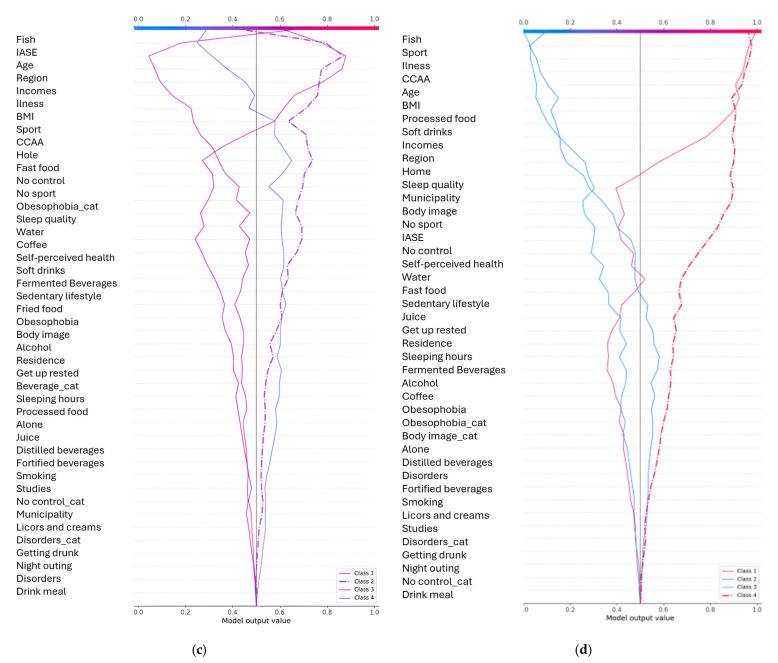
(**a**) Decision plot for instance 845. (**b**) Decision plot for instance 702. (**c**) Decision plot for instance 546. (**d**) Decision plot for instance 837.

Observation Analysis:

Observation 845 (Figure 6a): The model incorrectly predicts a vegan diet instead of the correct Mediterranean diet.

- The error arises primarily from the fried food feature, with subsequent features worsening the prediction.
- This is consistent with the confusion matrix (Figure 2), which shows poor accuracy for classes 2 (Mediterranean), 3 (vegan), and 4 (vegetarian).

Observation 702 (Figure 6b): The model correctly predicts a Mediterranean diet.

- Initially unsure, the model gains confidence as important features (e.g., fish) are analyzed.
- Fish is a key feature, splitting positive predictions for classes 1 and 2 and negative predictions for classes 3 and 4.

Observation 546 (Figure 6c): The model exhibits uncertainty when predicting intermittent fasting diet.

- The initial prediction seems correct, but uncertainty arises with the fish feature.
- This individual likely has a confusing diet (e.g., doesn’t eat fish but consumes junk food), making classification challenging.

Observation 837 (Figure 6d): The model predicts a vegetarian diet but shows difficulty distinguishing between Mediterranean and vegetarian diets.

- Certain features (e.g., fish) exponentially increase the probability of a Mediterranean diet, suggesting overlapping characteristics between classes 1 (Mediterranean) and 4 (vegetarian).
- The model struggles with nuanced differences, despite being confident about the prediction.

Key findings:

- The model performs well for simpler cases (e.g., Observation 702) but struggles with overlapping or contradictory dietary patterns.
- Fish, fried food, and class-specific features strongly influence predictions, often determining class distinctions.
- Misclassifications reveal areas where the model could be improved, particularly for better differentiation among classes 2, 3, and 4.

#### 3.3.2. Individual Conditional Expectation Plots

To scrutinize the connection existing amongst salient features and the predictions for different classes, Individual Conditional Expectation (ICE) plots were produced. Each of these plots served to illustrate individual predictive outcomes (depicted via blue lines) alongside the average influence (a red line, also known as the PDP) across the three distinct classes under consideration. The subsequent analysis brought to light discernible trends unique to each feature examined.

Concerning the intake of soft drinks in Figure 5a, a pronounced differentiation between the classes was evident. Class 1, for instance, displayed a positive correlation as the level of consumption went up, whereas Classes 0 and 2 presented relationships that were, conversely, negative. This observation rather strongly implies that the consumption of soft drinks acts as a noteworthy factor in distinguishing between these classes.

The categories pertaining to special diets in Figure 5b revealed transitions that were quite abrupt, most notably within Class 1. This class exhibited a conspicuous surge in probability around the 0.6 threshold mark. Classes 0 and 2, in contrast, showed inverse relationships, thereby suggesting that restrictions in one’s diet exert a considerable influence on how class assignments are made.

Patterns in fish consumption in Figure 5c displayed a noticeable degree of variation across the different classes. Class 1, for example, showed a tendency to decline as consumption increased, whilst Class 2 initially showed an increase before its probabilities became more stable. Class 0, on the other hand, maintained probabilities that were consistently low, which hints at fish consumption playing a role in helping to tell apart the different dietary patterns.

In comparison to some of the other variables, BMI in Figure 5d exhibited relationships that were more gradual and continuous in their nature. Class 1 managed to maintain the highest probabilities, even though there was a slight decrease observed at the higher end of the BMI values. Class 2 demonstrated a level of stability that was moderate, and Class 0 remained consistently at the lower end of the probability spectrum. This suggests that BMI, whilst subtle, exerts a persistent influence on which class an individual might belong to.

When looking at age patterns in Figure 5e, distinct characteristics for each class became apparent. Class 2, notably, showed an increase in probability within the older age brackets (around the 70th to 80th percentile). Class 1’s probabilities remained fairly stable until they started to decline in the more advanced age groups, whilst Class 0 showed only minimal variation, with perhaps a slight peak occurring during middle age.

Across all of the features that were examined, the considerable degree of variation seen in individual predictions when compared to the average effect does point towards significant interaction effects occurring between the variables. This suggests that the impact any given feature has on predicting class membership is, in fact, modulated by the other variables present within the model. This intricate interplay amongst the features serves to demonstrate the multifaceted character inherent in the classification of dietary patterns.

#### 3.3.3. Discovering the Typical Prediction Paths

**Figure 7:**
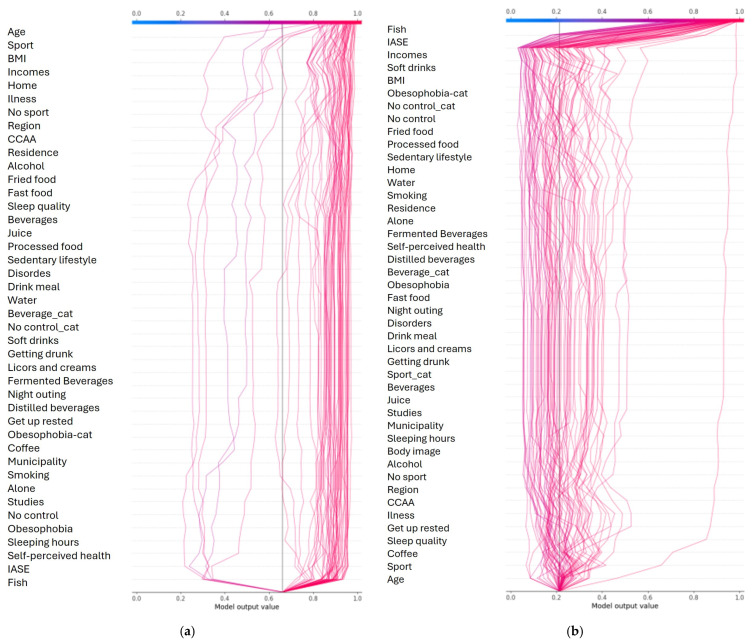

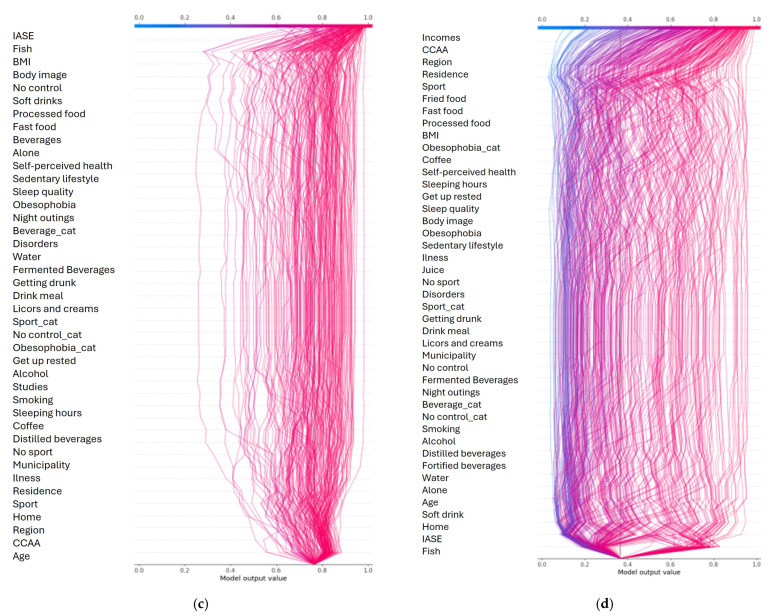
(**a**) Typical prediction path for a correct prediction of class 2. (**b**) Typical prediction path for a correct prediction of class 3. (**c**) Typical prediction path for a correct prediction of class 4. (**d**) Typical prediction path for a wrong prediction of class 1.

Given that the model struggles to accurately predict classes 2, 3, and 4, it is worth examining the typical prediction paths of correct predictions for these classes and the wrong predictions for class 1. Figure 7a shows a typical path for a correct prediction of class 2, while Figure 7b displays one for class 3. Figure 7a,c follow expected patterns, but Figure 7b (vegan diet) is unusual, as the model fails to recognize vegans who eat fish. Correct predictions tend to start with low confidence but become certain once IASE and Fish values are considered, confirming their importance. An outlier is observed in Figure 7d, where the typical pattern is unclear, but it is evident that fish and IASE are crucial, with most incorrect predictions having low fish values.

#### 3.3.4. Summary of Main Results

This experiment identifies fish as the most critical feature, effectively dividing diets into classes 1–2 (Mediterranean and intermittent fasting) and 3–4 (vegan and vegetarian). IASE also emerges as influential but does not interact with other features, suggesting it contributes to predictions independently.

Other features have varying degrees of influence:

- Age: Positively impacts Mediterranean diet predictions as it increases, and negatively impacting vegan and vegetarian diets as it decreases.
- Junk food: Positively influences classes 1 and 2 (Mediterranean and intermittent fasting).
- BMI: Vegetarians tend to have lower BMI, though the causal relationship remains unclear.
- Sport: Particularly significant for classes 2 (intermittent fasting) and 4 (vegetarian), often correlating with age.

The model performs reliably for predicting the Mediterranean diet but struggles with accuracy for the other three classes, as illustrated in the decision plots (Figure 3).

### 3.4. Interaction Between the Variables IASE and Fish

**Figure 8:**
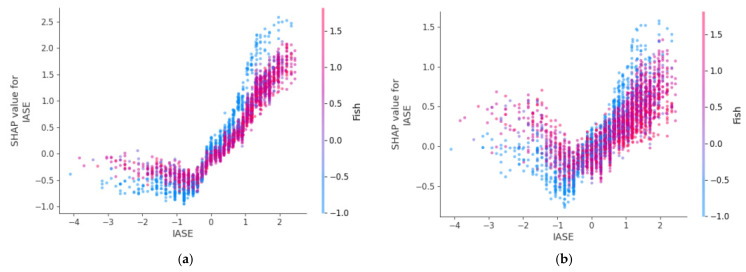

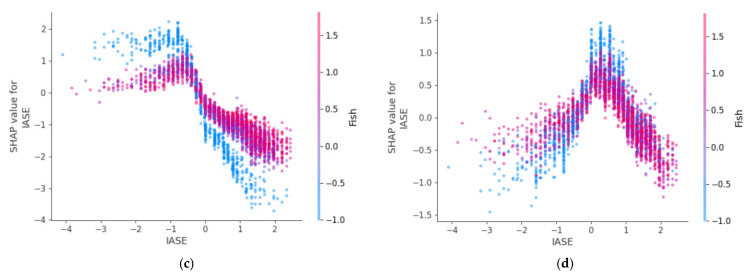
(**a**) IASE and fish interaction for class 0. (**b**) IASE and fish interaction for class 1. (**c**) IASE and fish interaction for class 2. (**d**) IASE and fish interaction for class 3.

A comparative analysis was conducted on two variables with the greatest impact on diet type prediction: the Healthy Nutrition Index (IASE) and fish consumption. The results found were:

Mediterranean diet (Class 0, Figure 8a):

- A strong correlation exists between IASE and fish consumption.
- Low IASE values correspond to low fish consumption, but as IASE increases, there is a clear turning point where higher IASE values lead to increased fish consumption.

Intermittent fasting diet (Class 1, Figure 8b):

- The correlation between IASE and fish is weaker compared to the Mediterranean diet.
- While similar trends are observed, greater data dispersion indicates less consistency in the relationship.

Vegan diet (Class 2, Figure 8c):

- As IASE scores increase, fish consumption nearly disappears.
- This pattern aligns with the vegan diet’s exclusion of all animal proteins, including fish.

Vegetarian diet (Class 3, Figure 8d):

- A complex relationship emerges.
- Initially, higher IASE scores correlate with increased fish consumption. However, fish intake then drops drastically, mimicking the vegan diet’s behaviour (Figure 8c).
- The red dots (indicating correct classifications) highlight this shift, suggesting that as IASE increases, vegetarian diets align more closely with vegan patterns regarding fish consumption.

This analysis highlights distinct relationships between IASE and fish consumption across dietary classes. While the Mediterranean and intermittent fasting diets show positive trends, the vegan and vegetarian diets reveal exclusionary patterns with increased IASE, reflecting their respective dietary restrictions. The vegetarian diet’s unique shift emphasizes its nuanced relationship with fish consumption.

## 4. Discussion

The data have shown that fish consumption is the most powerful indicator for distinguishing one type of diet from another. This finding is particularly relevant in the Spanish context, where the Mediterranean diet is traditionally prevalent. Fish is a key source of essential nutrients, such as omega-3 fatty acids, high-quality proteins, and various vitamins and minerals (Ali et al., 2022), which significantly contribute to cardiovascular health, brain development, and overall well-being (DiNicolantonio & O’Keefe, 2020). Although its consumption has been decreasing in recent years (Abellán Alemán et al., 2016), fish occupies a central place in the Mediterranean diet due to Spain’s geography as a peninsula surrounded by the Mediterranean Sea and the Atlantic Ocean, facilitating access to a wide variety of fresh fish and seafood. This availability has influenced the regular inclusion of fish in daily meals, making it a cornerstone of the region’s diet (Moreiras & Cuadrado, 2001).

Conversely, in vegan diets and many variants of vegetarian diets, fish is absent. These diets are characterized by the total exclusion of animal products (in the case of vegan diets) or the exclusion of meat and fish, with possible inclusions of dairy products and eggs (in some vegetarian variants) (Hargreaves et al., 2023). The absence of fish in these diets necessitates finding nutritional alternatives that can provide the same benefits that fish offers in the Mediterranean diet (Bali & Naik, 2023).

This contrast underscores the importance of fish consumption not only as a distinguishing element among different dietary patterns but also as a crucial component for ensuring balanced and healthy nutrition (DiNicolantonio & O’Keefe, 2020). Additionally, this analysis highlights the need to consider the nutritional and health implications when choosing and promoting different types of diets, especially in specific geographical and cultural contexts.

Another significant finding from the data is that younger individuals tend to adopt vegan or vegetarian dietary patterns more frequently, while older individuals are more likely to follow a classic Mediterranean diet. This generational difference in dietary preferences can be attributed to several interrelated factors. Firstly, the influence of celebrities, influencers, and activists promoting vegan and vegetarian lifestyles plays a significant role in the adoption of these diets (Pilař et al., 2021). Younger people are more exposed to and influenced by these information agents (Goodyear et al., 2018). Additionally, greater access to technology and social media among the youth facilitates their awareness of the benefits of plant-based diets, thereby motivating them to follow these diets. Conversely, older individuals have grown up in a culture where the Mediterranean diet has been the norm. This dietary pattern, deeply rooted in the traditions and culinary practices of the region, has demonstrated numerous health benefits (Guasch-Ferré & Willett, 2021; Shen et al., 2015; Dominguez et al., 2023). Adherence to this diet among older people may stem from a sense of cultural continuity and confidence in its proven benefits. Differences in the availability and cost of food also influence dietary choices. Fresh and local products that are part of the Mediterranean diet are easily accessible and affordable in many regions of Spain, particularly for older generations who may have more time to prepare home-cooked meals (Tsofliou et al., 2022).

Lastly, a significant factor impacting dietary choices is environmental and animal welfare concerns (Hopwood et al., 2020). Studies have shown that the production of meat and animal products is less sustainable and often does not prioritize animal welfare (Font-I-Furnols, 2023). In response to these environmental concerns, many young people adopt vegan and vegetarian diets, which are considered more sustainable (Mathur et al., 2020; Sabaté & Soret, 2014).

The results also show that people who follow a vegan and vegetarian diet tend to eat less “junk food” compared to those who follow a traditional dietary pattern. To explain this trend, one could point first to a practical fact: many fast food and junk food options contain animal products (Fuhrman, 2018), which limits the available choices for vegans and vegetarians, prompting them to seek healthier and less processed alternatives.

Additionally, this trend can be linked to the values and motivations that often accompany vegan and vegetarian dietary choices (Mathur et al., 2020; Sabaté & Soret, 2014). Sustainability and environmental concerns frequently extend beyond diet alone and translate into a more holistic approach to a healthy and sustainable lifestyle. Avoiding junk food, which typically has a high environmental impact due to its production methods, aligns with these values.

As regards to the tendency found on the practice of physical activity, significant differences are shown in the time devoted to physical activity per week between people who practice intermittent fasting and those following other diets, particularly the Mediterranean one. This is not surprising, as individuals who practice intermittent fasting are often more focused on weight control, and exercise can help achieve this goal (Welton et al., 2020). People who follow diets other than the Mediterranean one tend to be more concerned about their health and fitness.

Another noteworthy result obtained from the data analysis is that Catalonia and Galicia show higher adoption of plant-based diets compared to other parts of Spain, while the region of Murcia has a higher adherence to the practice of intermittent fasting. The prevalence of different diets in Spain varies due to regional, cultural, economic, and environmental factors influencing food preferences (Gherasim et al., 2020). Catalonia and Galicia show higher adoption of PBDs, possibly due to long-standing culinary traditions featuring plant-based dishes and seafood (Arija et al., 2019; Trabazo et al., 2019). Environmental awareness and access to fresh produce further support PBD adoption in these regions (Dixon et al., 2023; Nelson et al., 2016). The higher incidence of intermittent fasting in Murcia may stem from its culinary culture emphasizing fresh and healthy foods, potentially influencing perceptions of a balanced diet (Jiménez Monreal et al., 2019). Sampling bias, such as influencer promotion, could also impact reported diet trends in specific regions.

A consideration should also be made regarding the trend reflecting the healthy nutrition index with respect to the type of diet adopted. The IASE score reveals lower values for plant-based diets compared to the Mediterranean diet. This disparity can be attributed to the index’s calculation, which emphasizes a diverse diet including animal proteins like meat and dairy (Norte Navarro & Ortiz Moncada, 2011). Since vegan and raw vegan diets exclude these food categories, their IASE scores are significantly lower in these cases. Therefore, for this study, the IASE score may not accurately reflect the health benefits associated with these specific diets.

Concerning the presence of diagnosed eating disorders and the type of diet practiced, the slight inclination toward experiencing some form of eating disorder among individuals following diets other than the traditional Mediterranean diet, as indicated by the results, underscores dieting as one of the contributing factors to these disorders (Hetherington, 2000).

Finally, the CatBoost model achieved an acceptable overall accuracy of 87%, primarily due to the strong representation of the Mediterranean diet class. However, the macro-average precision (0.53), recall (0.47), and F1-score (0.48) indicate moderate performance across all classes, suggesting that the current features do not sufficiently capture the non-linearities and nuances necessary for accurately distinguishing specific dietary patterns. This moderate performance is particularly evident in the poor results for intermittent fasting, vegan, and vegetarian classes, where the model struggles due to insufficient training data.

### Strengths and Limitations

This study has several strengths, notably its large sample size (N = 22,181), which provides a comprehensive overview of health behaviours among young Spaniards. Geographical inclusivity is another strength, with data collected from all regions of Spain, including urban, rural, and island areas. The study’s breadth of variables allows for an in-depth exploration of how various nutritional, physical activity, social, and lifestyle factors interact with socio-demographic determinants, offering valuable insights into the health status of the Spanish population.

However, the study is not without limitations. Data collection through online surveys may introduce response bias, although efforts were made to mitigate this by using a closed and anonymous questionnaire format. Additionally, physical dissemination methods were employed to reach those not active on social networks. Self-report bias in nutritional surveys is a concern, as participants may provide inaccurate or biased information about their dietary habits. Despite these challenges, the study’s findings align with previous research, enhancing confidence in its conclusions. Finally, there was a gender bias observed, with 80.8% of respondents being female, reflecting a broader trend of male underrepresentation in surveys. Despite efforts to recruit male participants, achieving gender parity remained challenging, though a substantial sample of 4251 males was included in the study.

## 5. Conclusions

This study has shown that the strongest indicator for diet choice is fish consumption, with vegans abstaining entirely and some vegetarians consuming it occasionally, as it is normal for these dietary patterns. Other strong indicators include age, with younger individuals leaning towards vegan and vegetarian diets, and older people adhering to a traditional Mediterranean diet. Vegans and vegetarians consume less junk food and those who engage in regular sports often follow an intermittent fasting diet. Potential areas for further exploration include the impact of sleep quality and duration on diet choice, and regional dietary tendencies, such as why Catalans prefer vegan/vegetarian diets.

The model excels in predicting adherence to the Mediterranean diet but is less accurate for other diet types. This discrepancy stems from the unbalanced distribution of individuals across dietary classes, with a high number of Mediterranean diet followers and fewer adherents to other patterns. A more extensive study including a larger population of individuals following plant-based diets or intermittent fasting would be valuable for a detailed examination of the determinants of these diets.

Although it requires a very large sample of individuals, Explainable Artificial Intelligence, particularly the CatBoost method combined with the SHAP approach, can be extremely useful for detailed population behaviour analysis. This technique helps identify the most determinant variables and is likely to see increased application in the field of health sciences.

## Figures and Tables

**Table 1:** SMOTENC classification report.

Class	Precision	Recall	F1-Score	Support
1. Mediterranean diet	0.91	0.96	0.93	3248
2. Intermittent fasting	0.26	0.09	0.13	166
3. Vegan	0.47	0.46	0.46	70
4. Vegetarian	0.47	0.36	0.4	253
Accuracy			0.87	3737
Macro average	0.53	0.47	0.48	3737
Weighted average	0.84	0.87	0.85	3737

**Table 2:** Socio-demographic characteristics of sample (N = 22,181).

		Mean; SD or N (%)	
Male		4251 (19.2%)	
Female		17,930 (80.8%)	
Age (years)		34.9; 11.7	range (18–89)
Male Age		36.5; 13.4	range (18–84)
Female Age		34.5; 11.2	range (18–89)
	Total		
Age	N (%)		
Young (18–30)	9692 (43.7%)		
Middle Age (31–50)	9913 (44.7%)		
Adults (>50)	2576 (11.6%)		
Level of education			
Basic	7027 (31.7%)		
Higher	15,154 (68.3%)		
Income level			
Low	9727 (43.8%)		
Medium–high	10,616 (47.7%)		
Do not know/no answer	1838 (8.3%)		
City size			
<2000	1014 (4.6%)		
2000–10,000	3587 (16.2%)		
>10,000	17,580 (79.3%)		

Note: SD = standard deviation.

## Data Availability

The data presented in this study are available upon reasonable request to the corresponding author.

## Appendix A

**Table A1:** List of variables analysed and their typology.

Variables	Typology	
Sex	Categorical	Demographical features
Age	Numerical	
Municipality	Categorical	
Region	Categorical	
CCAA (Autonomous Communities)	Categorical	
Residence	Categorical	
Studies	Categorical	
Income	Categorical	
Home	Categorical	
Alone	Categorical	
Obesophobia	Categorical	Eating habits
No control	Categorical	
Body image	Categorical	
Fried Food	Categorical	
Fast Food	Categorical	
Ultra Processed Food	Categorical	
Fish	Categorical	
Water	Categorical	
Soft drinks	Categorical	
Juice	Categorical	
Coffee	Categorical	
Drink meal	Categorical	
Fermented beverages	Categorical	
Distilled beverages	Categorical	
Fortified beverages	Categorical	
Liquors and creams	Categorical	
IASE	Numerical	
Sedentary lifestyle	Categorical	Lifestyle
Sport	Categorical	
Reasons No Sport	Categorical	
Sleeping hours	Categorical	
Getting up rested	Categorical	
Sleep quality	Numerical	
Smoking	Categorical	
Night outings	Categorical	
Alcohol	Categorical	
Getting drunk	Categorical	
BMI	Numerical	Health
Self-perceived health	Numerical	
Diagnosed Eating Disorders	Categorical	
Illness	Categorical	
